# Persistence of dysfunctional immune response 12 months after SARS-CoV-2 infection and their relationship with pulmonary sequelae and long COVID

**DOI:** 10.1186/s12931-025-03200-1

**Published:** 2025-04-17

**Authors:** Tamara Cruz, Núria Albacar, Estibaliz Ruiz, Gema M. Lledo, Lídia Perea, Alba Puebla, Alejandro Torvisco, Núria Mendoza, Pau Marrades, Jacobo Sellares, Alvar Agustí, Odette Viñas, Oriol Sibila, Rosa Faner

**Affiliations:** 1 Institut d’Investigacions Biomèdiques August Pi i Sunyer (IDIBAPS), C/Roselló 149, 08036 Barcelona, Spain; 2 Centro de Investigación Biomédica en Red de Enfermedades Respiratorias (CIBER), Madrid, Spain; 3 Respiratory Institute, Hospital Clinic, C/Villaroel 170, 08036 Barcelona, Spain; 4 Immunology Department, Hospital Clínic, C/Villaroel 170, 08036 Barcelona, Spain; 5 Department of Autoimmune Diseases, Hospital Clínic, Barcelona, Spain; 6 Department of Biomedicine, Immunology Unit, University of Barcelona, Barcelona, Spain

**Keywords:** COVID-19, Pulmonary Sequelae, Long COVID, Persistence of viral reservoirs, Autoantibodies, Organ-damage markers, 1 year of convalescence, Biomarkers

## Abstract

**Introduction:**

Most patients recover fully after an acute infection by SARS-CoV-2. Some, however, may develop pulmonary *sequelae* (PS) and/or long COVID (LC). However, whether these two clinical conditions have similar or different pathogenic mechanisms is unknown.

**Methods:**

The levels of autoantibodies and 184 inflammatory and organ damage associated proteins in plasma were determined (by immunofluorescence and Olink panels, respectively) 1 year after an acute infection by SARS-CoV-2 in 51 patients with PS (DLCO < 80% ref), 31 patients with LC and 31 patients fully recovered (Rec). PS was defined by the presence of reduced carbon monoxide diffusing capacity (DLCO) lower than 80% ref. LC was defined by the presence of chronic symptoms in the absence of an alternative diagnosis.

**Results:**

We found that patients with PS or LC both showed increased levels than Rec of anti-microbial, immune cell activation and recruitment related proteins. Patients with PS showed higher levels of anti-nuclear autoantibodies, whereas LC patients had increased levels of organ-damage associated proteins. In patients with PS most of the elevated proteins correlate with the impairment of lung function (DLCO). Finally, in PS we additionally performed the determinations at an earlier time point (6 months) and showed that the expression of CCL20 and IFN-ɣ was already higher at 6 months, while CCL3 and CCL19 increase from 6 to 12 months, suggesting a pathogenic role in PS persistence.

**Conclusions:**

Patients with PS or LC have abnormal but different persistent circulatory immune and organ damage biomarkers, suggesting different underlying biology of both post-COVID conditions.

**Supplementary Information:**

The online version contains supplementary material available at 10.1186/s12931-025-03200-1.

## Introduction

After an acute infection of SARS-CoV-2 most patients recover fully. Yet, some patients develop structural and/or functional pulmonary *sequelae* (PS) [1] whereas others experience long-term symptoms, including fatigue, chest pain, cough, neurocognitive alterations and/or muscle and joint pain without a discernible diagnosis (long-COVID—LC) [2, 3]. Whether the biologic mechanisms underlying PS and LC are similar or different is unclear. Previous studies have shown that PS is associated with systemic endothelial dysfunction markers (VEGF-A, sITGaM and sITGb2) [4], mitochondrial dysfunction [5], inflammation (IL-1α and TGF-β) [6], neutrophil activation (LCN2) [7], and fibrotic markers (MMP-7 and HGF) [8], but the role of these biologic mechanisms in LC is unclear. On the other hand, LC has been shown to be associated with autoimmunity abnormalities, including cross-reactive T-cells, antigen-specific tissue resident memory cells, and autoantibodies [9]. Higher titers of autoantibodies during the acute COVID-19 infection had been associated with the development of PS at 3 months follow-up [10], but the persistence of autoantibodies has not been explored in PS. Recent findings in LC patients 1 year after the acute episode point towards a persistent inflammation (i.e. complement and coagulation alterations) and tissue injury markers [11]. Finally, both PS and LC have been associated with an increased SARS-CoV-2 specific T cell response with an exhausted phenotype (CD3+CD8+CD28−), suggesting a non-resolved, persistent, adaptive response potentially driven by persistence of viral reservoirs in some organs [12, 13]. In this study, we sought to determine the inflammatory immune response, autoimmunity and host damage response to contrast the similarities, and differences in patients with PS and LC vs. patients who recovered fully (Rec) 1 year after the acute SARS-CoV-2 infective episode.

## Methods

### Study design, patients and ethics

This is a prospective, observational, controlled study that included 113 adults who were hospitalized in our institution between May and November 2020 because of PCR-confirmed COVID-19 pneumonia (Table 1). The severity of the acute disease was determined according to the seven-category severity scale recommended by WHO [14]. During hospitalization patients were treated according to international recommendations [15]. All the participants were unvaccinated at the time they experienced the acute COVID-19 episode. The study participants were divided into two groups: (1) patients with respiratory symptoms and (2) patients suspected of Long-COVID (LC). After discharge, patients with respiratory symptoms were followed up according to the Spanish Society of Pulmonology and Thoracic Surgery (SEPAR) for post-COVID-19 patients [16]. These patients were visited in the outpatient clinic at 3, 6, 9 and 12 months (± 2 months). In parallel, in a second group of patients, with complains of fatigue, chest pain, arthralgia, myalgia, headache, neurocognitive dysfunction or autonomic dysfunction, follow-up was performed due to possible long-COVID.

**Table 1:** Main clinical characteristics of the enrolled patients

	All	Rec	PS	p.val	LC	p.val	p.val
	N = 113	N = 31	N = 51	PS vs Rec	N = 31	LC vs Rec	PS vs LC
Age	57.2 (13.3)	57.4 (13.3)	59.6 (12.6)	0.464	51.7 (13.6)	0.127	**0.023**
Gender (m)	50 (47.6%)	11 (35.5%)	24 (47.1%)	0.425	15 (65.2%)	0.059	0.232
Previous comorbidities							
Cardiovascular	36 (32.8%)	7 (22.6%)	25 (49.0%)	**0.032**	4 (12.9%)	1	**0.001**
Autoimmune	15 (23.4%)	3 (12.5%)	9 (22.5%)	0.483	3 (37.5%)	0.203	0.51
Connective tissue disease	3 (4.7%)	1 (4.17%)	2 (5.13%)	1	0 (0.00%)	1	1
Obesity	17 (25.4%)	5 (20.8%)	9 (23.1%)	1	3 (33.3%)	0.822	0.974
DLCO 12 m	78.9 (14.5)	91.1 (10.1)	68.4 (9.53)	**< 0.001**	89.1 (7.04)	0.637	**< 0.001**
PS 12 m, n (%)	51 (52.0%)	0 (0.00%)	51 (100%)	**< 0.001**	0 (0.00%)		**< 0.001**
DLCO 6 m	80.4 (17.4)	92.8 (15.8)	71.7 (12.5)	**< 0.001**	82.2 (19.5)	0.164	0.152
PS 6 m, n (%)	40 (46.5%)	4 (12.9%)	33 (71.7%)	**< 0.001**	3 (33.3%)	0.316	0.051
Non-pulmonary symptoms 12 m	60 (53.1%)	0 (0.00%)	29 (56.9%)	**< 0.001**	31 (100%)	**< 0.001**	**< 0.001**
Non-pulmonary symptoms 6 m	74 (64.9%)	14 (44.0%)	52 (71.4%)	**0.033**	31 (100%)	**0.001**	**0.03**
ICU admission, n (%)	42 (39.3%)	13 (41.9%)	25 (49.0%)	0.693	4 (16.0%)	0.071	**0.011**
Corticoids during acute episode	37 (57.8%)	12 (54.5%)	23 (63.9%)	0.668	2 (33.3%)	0.648	0.202
Severity (WHO)				0.386		**< 0.001**	**< 0.001**
1	27 (23.9%)	1 (3.23%)	6 (11.8%)		20 (64.5%)		
2	1 (0.88%)	0 (0.00%)	1 (1.96%)		0 (0.00%)		
3	20 (17.7%)	8 (25.8%)	9 (17.6%)		3 (9.68%)		
4	17 (15.0%)	5 (16.1%)	8 (15.7%)		4 (12.9%)		
5	21 (18.6%)	8 (25.8%)	10 (19.6%)		3 (9.68%)		
6	8 (7.08%)	1 (3.23%)	7 (13.7%)		0 (0.00%)		
7	11 (9.73%)	3 (9.68%)	7 (13.7%)		1 (3.23%)		

Results are expressed as mean and standard deviation. The comparison was made using a Mann–Whitney test between the PS and the LC groups with the recovered group

Statistically significant comparisons (*p* < 0.05) are highlighted in bold

In this study, long-COVID was defined as the persistence (> 2 months) of symptoms 3 months after the onset of COVID-19 that could not be explained by an alternative diagnosis, thus excluded patients with impaired DLCO or with known previous diseases that could present similar symptoms.

In the current analysis, in order to address the unavoidable limitation that lung function tests had not been determined before hospitalization because of the acute COVID-19 episode, only patients without any previously known pulmonary disease that could affect the levels of DLCO were included in the study (only 4 patients with asthma).

Finally, patients were categorized into three groups based on their health status at 12 months after hospital discharge: 51 PS (defined by DLCO < 80% ref, with or without other non-pulmonary symptoms), 31 LC (defined by non-pulmonary symptoms and DLCO > 80% ref.) and 31 Rec (asymptomatic with DLCO > 80% ref). For the current determinations and analyses we used samples and data collected at 12 months and for the PS group we also performed a time-course using samples collected at 6 months.

### Measurements

#### Clinical and lung function parameters

Symptoms were evaluated at each clinical visit using SF-36 and Fatigue-structured questionnaires [2, 17]. Lung function (spirometry, DLCO) was also measured at each clinical visit following ERS/ATS standards using the Roca equations for reference values [18].

#### Blood sampling

Blood was collected by peripheral venipuncture in EDTA and Vacutainer SST advance tubes (Fisher Scientific, US) at 6 and 12 months after discharge. Blood tubes were centrifuged at 600 *g* during 10 min or 1000 *g* during 15 min (at 4 °C) to obtain plasma and serum, respectively. Samples were stored at −80 °C until analysis.

#### Detection of complement, immunoglobulins and autoantibodies at 12 months

Complement (C3, C4) serum levels and activity (CH50) were measured by turbidimetric immunoassay (Atellica CH C3 and Atellica CH C4; Siemens, New York, NY, USA and Autokit CH50; Fujifilm Wako Chemicals, Neuss, Germany, all are tested in Atellica CH Solution, Siemens, New York, NY, USA). Reference values were: 0.870–1.700 g/L for C3, 0.110–0.540 g/L for C4 and 28–60 U/mL for CH50. Hypocomplementemia was considered when either CH50 activity or C3 or C4 levels were lower than reference values. Immunoglobulin subtypes were measured also by turbidimetric immunoassay in the core facility of our hospital. Reference values were 6.8–15.3 g/L, 0.66–3.65 g/L and 0.36–2.61 g/L for IgG, IgA and IgM, respectively.

The presence of IgG autoantibodies was determined by indirect immunofluorescence assays (IFA) on HEp-2 cells and triple rat tissue (Werfen, US). Only positive results with a serum dilution higher than 1/80 were considered positive. Anti-nuclear and anti-cytoplasmic patterns of autoantibodies were reported, according to International Consensus on ANA Patterns (ICAP) nomenclature [19]. Additionally, IgG antigen-specific autoantibodies associated with systemic autoimmune diseases (autoantibodies anti-U1-RNP, Sm, Ro52, Ro60, SS-B, Scl-70, Jo-1, Cenp-B, and Ribosomal-P) were determined using the particle-based multi-analyte technology (PMAT) on the Aptiva instrument (CTD Essential Reagent Nº 723100, Werfen, US) and autoantibodies anti-IFNa and IFNw were quantified by Luminex as described before [20]. The number of total anti-nuclear auto-reactivities (ANAs) per patient was calculated by adding the number of patients positive for the ANA patterns AC1, AC4, AC5, AC6, AC8-9-10 and AC10, and for the anti-cytoplasmic patterns AC16, AC19, AC20 and AC21 was considered.

#### Inflammation and organ damage proteins

The relative abundance in plasma of 184 proteins was quantified with two Olink multiplex proximity extension assays (Inflammation and Organ Damage panels) [21]. A limit of detection (LOD) was established based on negative controls (included in each run). Biomarkers that did not pass quality control or the LOD were excluded from the analysis. ELISAS for Growth/differentiation factor 15 (GDF-15) and WAP Four-Disulfide Core Domain 2 (WFDC2) (R&D, US) were performed following manufacturing conditions.

#### Protein interaction and functional enrichment

The biological processes associated with differentially expressed proteins were computed using the Gene Ontology (GO) biological function and the KEGG pathways with ClusterProfiler [22]. To explore the interactions between proteins, the Search Tool for the Retrieval of Interacting Genes/Proteins (STRING) was used [23].

### Statistical analysis

Results are presented as n, frequency, mean ± standard deviation (SD) or median [interquartile range—IQR]. Groups were compared using Kruskal–Wallis with Wilcoxon pairwise post-hoc comparison and FDR adjustment, and Chi-square test, as appropriate. Bivariate correlations were analyzed using the Spearman's rank test. A p value < 0.05 was considered significant. Analyses were performed using R version 4.3.

## Results

### Patient characteristics

Table 1 presents the main characteristics of participants at the visit 6- and 12- months visits after hospital discharge. Age and sex were similar between PS and Rec groups, but LC patients were slightly younger and PS patients at baseline presented higher prevalence of previous cardiovascular disease. The COVID-19 severity score during the acute episode was lower in patients with LC than in PS or the Rec. Between 6 to 12 months after infection, the proportion of patients with non-pulmonary symptoms decreased from 64.9 to 53.1% (p = 0.22). Interestingly, up to 44% of the Rec (12 months) presented non-pulmonary symptoms at 6 months and 56.9% of patients with PS also reported non-pulmonary symptoms at 12 months. Non-pulmonary symptoms were considered only in the absence of previous related disease.

### Total immunoglobulin levels and presence of autoantibodies

The total dose of IgM and the IgG/IgA and IgM/IgA ratios were increased in LC compared to Rec while PS presented similar levels than Rec (Table 2). However, we did not find any statistically significant difference across the 3 study groups in the levels of specific autoantibodies and/or patterns, but the percentage of patients presenting any type of anti-nuclear reactivities were increased in PS compared with the Rec (23.5% vs 3.2% of Rec, Chi-square 0.033) and tended to be increased in LC patients (22.6% vs 3.2% of Rec, Chi-square 0.058), Supplementary figure S1 and Table 2.

**Table 2:** Immunoglobulin dosage, and presence of autoantibodies

	Rec (n = 31)	PS (n = 51)	PS vs Rec p-val	LC (n = 31)	LC vs Rec p-val	PS vs LC p-val
Immunoglobulin subtypes						
IgA (g/l)	2.38 [1.77;2.92]	2.11 [1.63;2.89]	0.586	1.85 [1.46;2.45]	0.092	0.229
IgG (g/l)	10.0 [8.30;11.5]	10.3 [9.00;12.7]	0.317	11.2 [9.25;12.6]	0.084	0.397
IgM (g/l)	0.96 [0.54;1.14]	0.89 [0.68;1.39]	0.456	1.08 [0.92;1.57]	**0.032**	0.182
IgG/IgM Ratio	11.0 [8.91;16.2]	11.6 [7.15;17.8]	0.800	10.4 [6.72;13.1]	0.257	0.393
IgG/IgA Ratio	4.68 [3.30;5.95]	4.71 [3.64;6.72]	0.371	6.51 [4.53;7.32]	**0.013**	0.062
IgM/IgA Ratio	0.48 [0.26;0.63]	0.40 [0.27;0.88]	0.506	0.63 [0.43;0.76]	**0.012**	0.196
ANAs in IF patterns (n and % of positive patients)						
Homogeneous (AC-1)	0	6 (11.8%)	0.122	3 (9.7%)	0.126	1
Fine speckled (AC-4)	1 (3.2%)	3 (5.8%)	0.989	3 (9.7%)	0.317	0.84
Large speckled (AC-5)	0	2 (3.9%)	0.705	3 (9.7%)	0.106	0.56
Multiple (AC-6)	0	3 (5.8%)	0.442	0	NA	0.44
Nucleolar (AC-8-9-10)	0	3 (5.8%)	0.442	2 (6.4%)	0.294	1
Punctate (AC-10)	0	0	NA	2 (6.4%)	0.272	0.27
Total anti-nuclear	1 (3.2%)	12 (23.5%)	**0.033**	7 (22.6%)	0.058	0.91
ANCAs in IF patterns (n and % of positive patients)						
Filamentous (AC-16)	0	0	NA	1 (3.2%)	0.8	0.8
Fine speckled (AC-20)	0	1 (2%)	1	0	NA	NA
AMA (AC-21)	0	1 (2%)	1	0	NA	1
Fine nailing	0	0	NA	1 (3.2%)	0.8	1
Total anti-cytoplasmic	0	2 (4%)	0.705	1 (3.2%)	1	1
Autoantibodies related with autoimmmune diseases (n and % of positive patients)						
Anti-CPG antibodies	1 (3.2%)	5 (9.8%)		1 (3.2%)	1	0.2
Anti-DFS70	6 (19.4%)	14 (27.5%)	0.574	4 (12.9%)	0.730	1
Anti-Ro60	2 (6.45%)	1 (1.96%)	0.657	1 (3.23%)	1.000	1
Anti-SSB	0	1 (1.96%)	1.000	0	NA	1
Anti-U1-RNP	5 (16.1%)	2 (3.92%)	0.131	2 (6.45%)	0.422	0.7
Anti-Scl70	0	2 (3.92%)	0.705	0	NA	1
Anti-CENP-B	0	2 (3.92%)	0.705	2 (6.45%)	0.472	0.66
Total anti-protein	10 (32.2%)	19 (31.4%)	0.825	10 (32.2%)	1.000	1
Total autoreactivities	12 (38.7%)	28 (55%)	0.232	14 (45.2%)	0.797	0.55

For immunoglobulin subtypes, results are expressed as median, 95% interquartile range, and compared to Rec with Mann–Whitney. For autoantibodies, the n and percentage of positive patients is presented, and were compared to Rec with a Chi-square. AC means Anti-Cell and is used by the International Consensus on ANA Patterns (ICAP) coding of HEp-2 IF patterns

Statistically significant comparisons (*p* < 0.05) are highlighted in bold

### Inflammation proteins

**Fig. 1:**
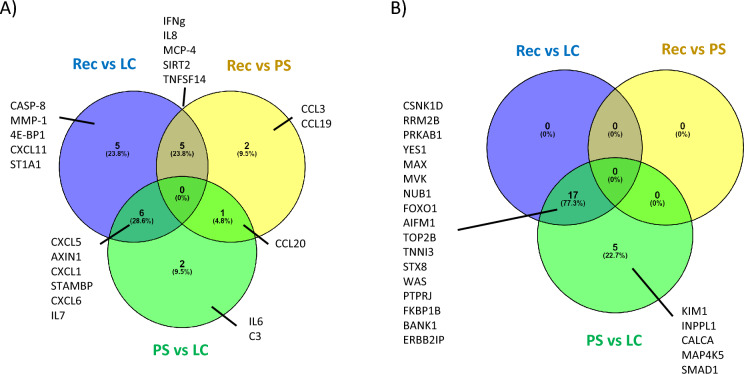
Inflammatory markers are increased in PS and LC while organ-damage proteins are only increased in LC. **A** Venn diagram showing the plasma inflammatory markers with different levels in the comparison between the three studied groups and **B** Venn diagram for the organ-damage related proteins

The levels of the complement protein C3 were elevated in patients with PS (vs. LC) (Supplementary table S1). From the 96 inflammatory proteins of the Olink panel, 21 were different across the study groups (Fig. 1A, Table S1). IFN-γ, IL-8, MCP-4, SIRT2, and TNFSF14 were elevated in both PS and LC vs. Rec, suggesting an ongoing IFN-γ and TNF-α response in both groups. On the other hand, IL-6 was higher in PS vs. LC. CCL20 was exclusively elevated in PS and CCL3 and CCL19 were increased in PS vs. Rec. CXCL5, AXIN1, CXCL1, STAMBP, CXCL6, and IL-7 were higher in LC than in both PS and Rec. The interaction network of the altered proteins in each comparison was represented in Supplementary figure S2.

### Correlation with DLCO and its evolution from 6 to 12 months in patients with PS

**Fig. 2:**
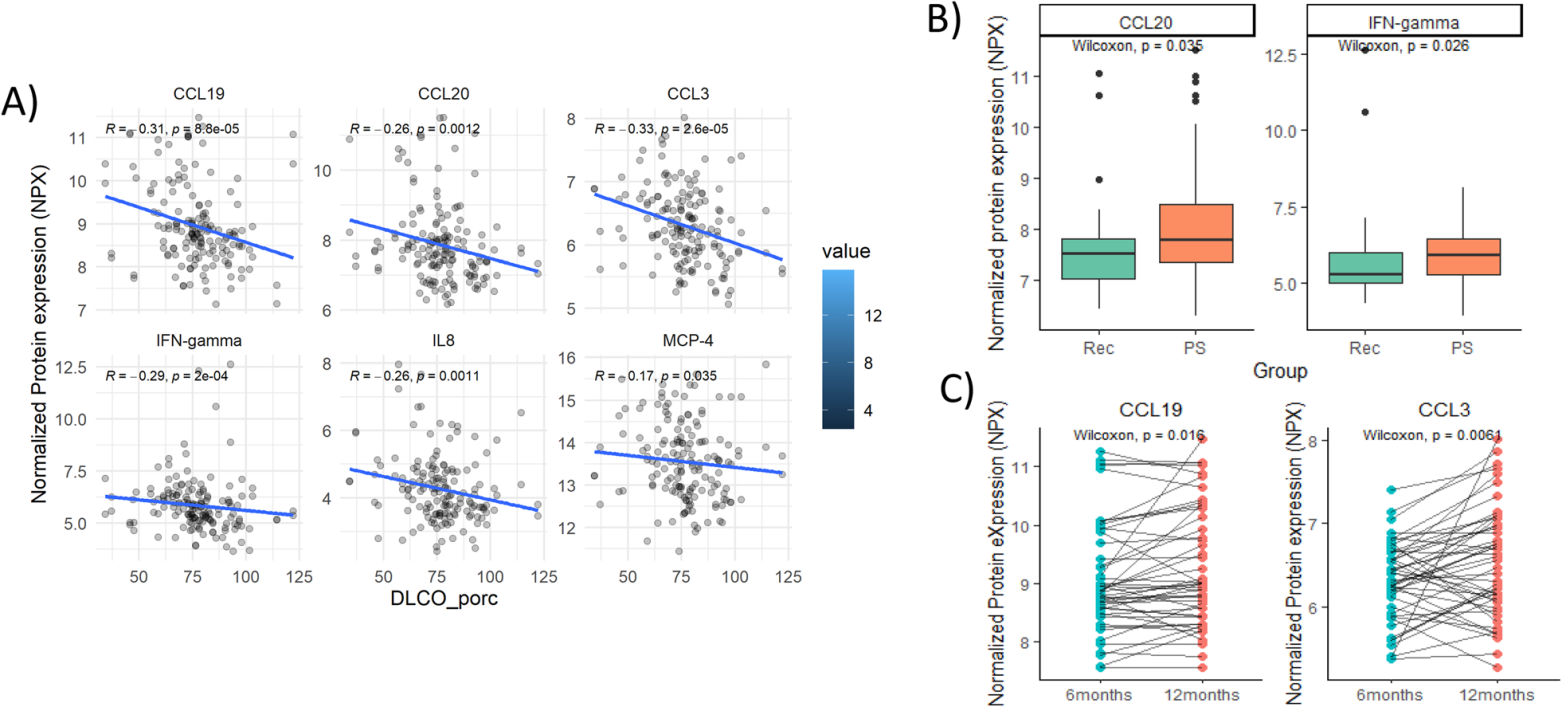
The inflammatory markers increased with severity of the PS and changes over time post-acute episode. **A** The levels of plasma inflammatory markers correlate with the severity of the PS by DLCO. **B** At 6 months post-infection PS patients had increased levels of CCL20 and IFN-ɣ. **C** There is an increased in the plasma inflammatory markers at 12 months post-discharge

Figure 2A shows that 6 out of the 8 cytokines with increased levels in PS (CCL19, CCL20, CCL3, IFN-γ, IL-8, MCP-4) negatively correlate with the level of DLCO at 12 months. Thanks to the design of our cohort with repeated visits, we could evaluate the levels of these mediators at an earlier time point (6 months). Interestingly, at 6 months only CCL20 and IFN-γ were increased in PS (Fig. 2B), suggesting a persistent elevation in PS, starting early post SARS-CoV-2 infection. Finally, we performed a paired t-test to study their change from 6 to 12 months. Figure 2C shows that CCL3 and CCL19 increased their levels from 6 to 12 months. All these results are summarized on Supplementary Table 2.

### Functional enrichment

**Fig. 3:**
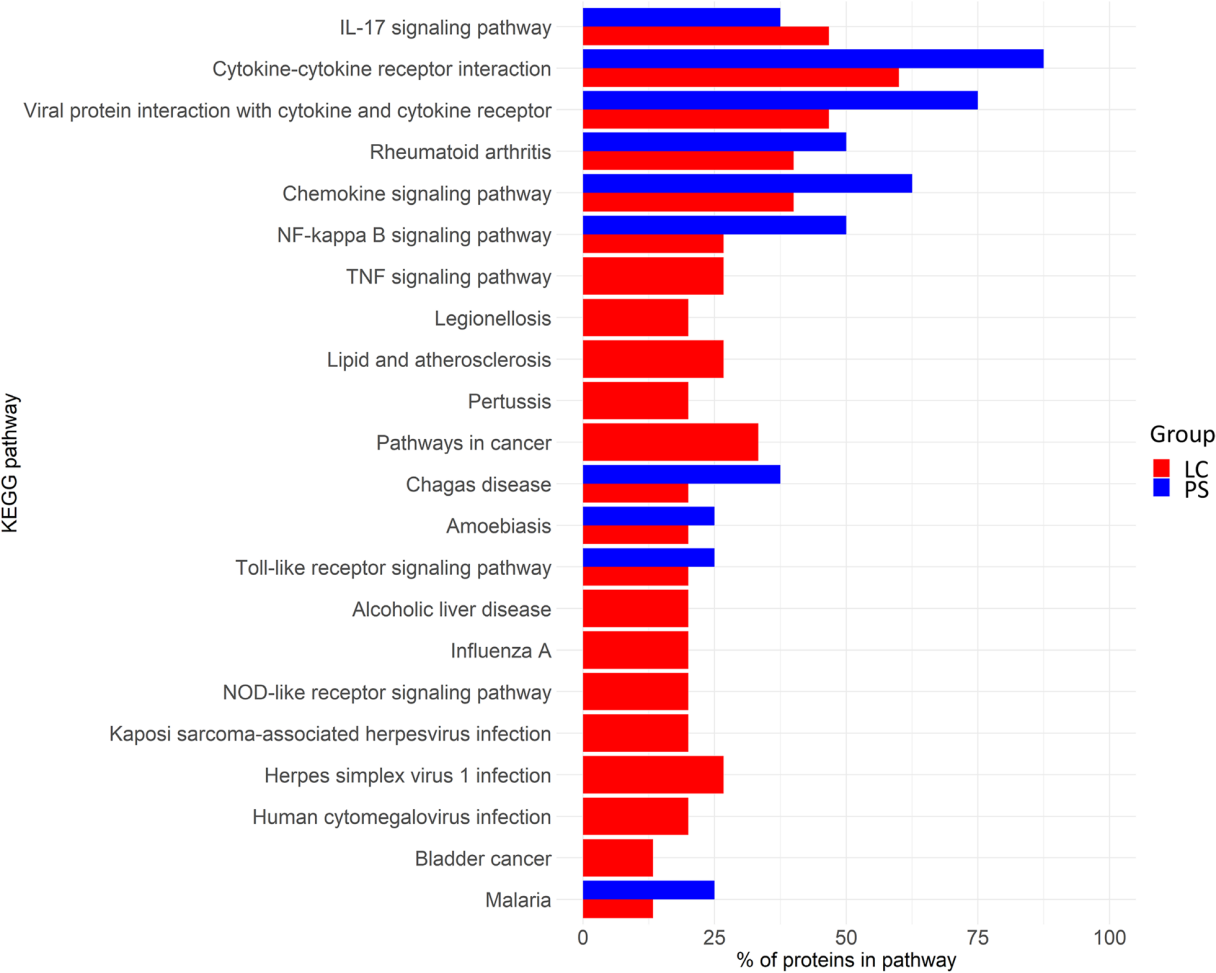
Biological pathways altered in patients with PS and LC. The percentage of plasma elevated proteins in PS or LC is represented for each KEGG pathway

Proteins increased (vs. Rec) in both PS and LC patients were involved in immune cell migration, activation, and anti-microbial immune response (Supplementary Table 3). Specifically, the altered pathways were IL-17, viral response, NF-KB signaling, and Toll-like receptors (TLRs) pathways (Fig. 3). In addition, PS patients showed functional enrichment related to T cells. Compared with LC, PS showed acute inflammation and tissue repair ontologies, whereas antimicrobial responses were increased in LC (Supplementary Table 4).

### Organ damage markers

**Fig. 4:**
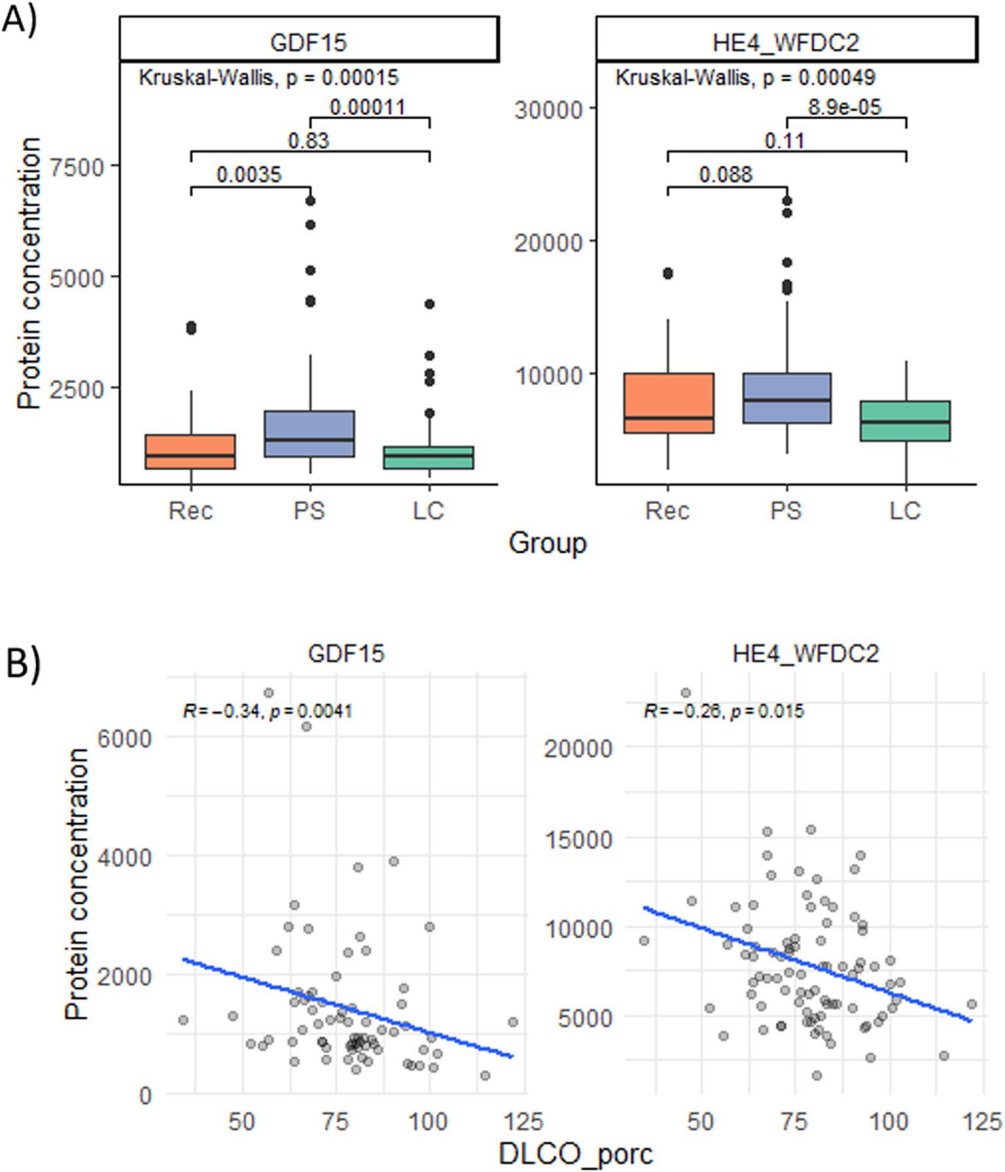
PS patients had increased levels of chronic lung disease associated proteins. **A** Boxplot of the plasma levels of GDF-15 and WFDC2. **B** Correlation plots of the levels of GDF-15 and WFDC2 and the 12 months DLCO

Circulating organ damage markers were similar in PS and Rec, but LC patients had 22 proteins with altered plasma levels (vs. PS), from which 17 were also increased vs. Rec (Fig. 1B and Table S5). All of them were increased in the LC group, except for TNNI3 and CALCA which were reduced. Some of the altered proteins had been associated with the acute COVID episode, cardiac injury or stress-response (Supplementary Table 6). Finally, the plasma levels of GDF15 and WFDC2 were measured due to their previous association with damage in several chronic lung diseases [24, 25]. Figure 4 shows that both proteins were elevated in patients with PS and that their levels correlated with the DLCO at 12 months.

## Discussion

The main and novel observation of this study is that 1 year after the acute COVID-19 episode, patients with both PS and LC have evidence of systemic inflammation but the former shows increased levels of C3 protein and higher percentage of patients presented anti-nuclear autoantibodies whereas the latter has increased circulating levels of organ damage markers.

### Previous studies

In contrast to our study, most post-COVID studies did not discriminate between PS and LC and were performed during the first months after the acute episode. A few ones studied PS 1 year after hospital discharge and found that MMP1, MMP7, periostin and VEGF levels were not increased in plasma [26] but, in keeping with our findings here, they also found increased C3 levels too [27]. Another study described NK cells with markers of impaired activation or exhaustion and reduced proportion of B cells [28]. In the BAL of patients with PS a previous study has shown antigen-specific memory B and T cells with dysregulated CD8 T cell responses [29]. All these alterations may contribute to the persistent systemic inflammation we found in our study in PS patients.

Studies in patients with LC studied at 1 year of convalescence showed increased levels of inflammatory markers [30], cytotoxic features [29] and autoantibodies [31]. The latter is at variance with our results here, but it is of note that controls in these studies were healthy, age-matched controls who never suffered COVID-19 and that higher autoantibody levels were observed at 3 months with progressive temporal reduction. A recent publication has described alterations in the complement cascade as the main driver of inflammation [11]. In this work the LC group had a severe acute episode of the disease and there is no division of the post-COVID patients based on pulmonary affectation. Our results go in line with this observation of elevated levels of the C3 complement in the group with a more severe acute disease, in our study the PS group. Another previous study proposed the stratification of LC patients into an inflammatory and a non-inflammatory type based on neutrophil activity, B cell memory alterations, and autoreactivity, but this study failed to identify an association of such a biological classification with any clinical characteristic of the patients [32]. A recent review described the effect of COVID-19 vaccination on reducing the risk to develop LC [33]. The patients of our cohort were recruited during the first year of the pandemic and thus were not vaccinated when they suffered the acute episode of the disease. Finally, the study with the longest convalescent period so far showed persistence of plasma metabolic alterations 2 years after the acute episode in patients with LC [34].

### Interpretation of novel findings

Total dose of IgM and the IgG/IgA and IgM/IgA ratios were increased in LC compared to Rec while PS presented similar levels to Rec, suggesting an active process in patients with LC. Additionally, both PS and LC patients presented elevated plasma levels of proteins implicated in anti-microbial immune response. This suggests that persistent viral reservoirs can be inducing a persistent inflammation in both PS and LC patients. Yet, both types of patients exhibited some noticeable differences.

On the one hand, several of the proteins increased in PS patients correlate negatively with DLCO level at 12 months, indicating that increased levels are associated with the severity of the lung impairment. Specifically, CCL3 and CCL19 were elevated at 12 months, but not at 6 months, suggesting that they are biomarkers of the mechanism driving the perpetuation of PS. Both CCL3 and CCL19 are chemokines, one responsible for polymorphonuclear cells and the latter of T and B cells migration to secondary lymphoid organs, suggesting that the adaptive immune response might have a role in the development of long term PS. In turn, CCL20 and IFN-γ were persistently elevated after SARS-CoV2 infection making them promising early biomarkers of PS development. PS patients also had increased levels of complement C3 suggesting an active immune process in this group. Finally, PS patients showed increased levels of GDF15 and WFDC2, both mediators of epithelial damage [35], associated with lung alterations and chronic lung diseases. GDF15 was associated with fibrotic interstitial lung diseases [35, 36] so it may be indicate fibrotic remodeling of the lung impairment. WFDC2 was elevated in PS and in the Rec, the groups with a more severe acute episode of COVID-19 and it had been recently described increased with the severity of the disease [37].

On the other hand, LC patients showed abnormal plasma levels of a range of cardiac and cellular stress markers (Table S5). Further, TNNI3 (Troponin I) myocardial protein and CALCA (calcitonin) were the only two proteins with reduced plasma levels in LC. Of note, both proteins have been associated with the severity and mortality of the COVID-19 acute episode [38]. These observations are consistent with the results in our cohort where their levels continue to be increased after 12 months of convalescence in Rec. and in PS, the groups with a more severe disease. Interestingly, FKBP1B and FOXO1 [39] are also related to cardiac function, but contrary to TNNI3 they are increased in the LC group, suggesting their potential involvement in the long-term cardiac dysfunction instead of the acute phase of the infection. Several of the proteins elevated in patients with LC have been previously associated with different SARS-CoV2 infective mechanisms, suggesting again the existence of a viral reservoir. Specifically, FKBP1B [40], PRKAB1 [41] and CSNK1D [42] have been associated with SARS-CoV2 viral replication, and KIM1 was described as a viral entry factor. SMAD1 [43] increases endothelial permeabilization and its inhibition in mice models of SARS-CoV2 infection showed a reduced mortality. FOXO1 [44] promotes airway inflammation and a knock-out mice model protected from severe COVID-19 inflammation disease. Remarkably, up to 13 of these proteins have immune-related functions. STX8 and YES1 are implicated in T-cell activation, MAP4K5, RRM2B, FOXO1, ERBB2IP and INPPL1 can promote inflammation and KIM1, MVK and BANK1 have been associated with autoimmune diseases. Collectively, these observations suggest a persistent antiviral response in both PS and LC, while PS also presents lymphocyte homing chemokines while in LC the biomarkers are related to cardiac and cellular organ damage.

### Strengths and potential limitations

The strengths of our study are the wide range of immune mediators and antibodies measured, the long-term follow-up period (12 months), the comparison of patients with PS, LC and Rec, and the exploration of the changes between 6 and 12 months of mediators associated to DLCO, that have not been addressed in previous studies. We acknowledge, however, that our sample size is limited, and that we studied only plasma samples, so our observations may not reflect the lung pathobiology in PS. Likewise, lung function was not determined before the acute COVID-19 episode but to address this unavoidable limitation we only included in our analysis patients without any previously known pulmonary disease affecting the DLCO. Patients with PS had increased cardiovascular comorbidity prior to the study entry, accordingly we cannot rule out that part of the non-pulmonary symptoms reported by these patients during follow-up are associated with these comorbidities. However, this doesn’t represent a confounding factor for the interpretation of the cardiac-related markers as alterations were found only on the LC group, PS presented similar levels to the Rec. Finally, to rule out the possibility of previous autoimmune diseases being a confounding factor for the result interpretation was address controlling the patients with previous autoimmune diseases to hypothyroidism (n = 15) with similar frequencies across the studied groups.

## Conclusions

Patients with PS or LC show persistent systemic inflammation 12 months after the acute COVID-19 episode, but CCL3 and CCL19 are specifically associated with PS, and the deterioration of DLCO in these patients, whereas LC patients present increased circulating levels of organ-damage markers. Collectively, these observations suggest a common persistent antiviral response with different underlying biology of both post-COVID conditions. Further studies are needed in order to understand whether these differences are host-specific or virus specific.

## Supplementary Information

Supplementary Material 1.

## Data Availability

All raw data generated during this study are available from the corresponding author on reasonable request. Tables with the full results of the analysis performed to support the conclusions are available in the main manuscript and the online supplement.

## Abbreviations

COVID: Coronavirus disease
PS: Pulmonary sequelae
LC: Long COVID
Rec: Recovered
DLCO: Carbon monoxide diffusing capacity
IFN: Interferon
VEGF-A: Vascular endothelial growth factor A
sITGaM: Integrin alpha M
sITGb2: Integrin beta 2
IL-1α: Interleukin 1 alpha
TGF-β: Transforming Growth Factor beta
LCN2: Lipocalin-2
MMP-7: Metalloproteinase 7
HGF: Hepatocyte Growth Factor
WHO: World Health Organization
SEPAR: Spanish Society of Pulmonology and Thoracic Surgery
ERS/ATS: European Respiratory Society/American Thoracic Society
Ig: Immunoglobulin
IFA: Immunofluorescence assays
ANA: Anti-Nuclear Autoreactivities
PMAT: Particle-based multi-analyte technology
LOD: Limit of detection
GDF-15: Growth/differentiation factor 15
WFDC2: WAP Four-Disulfide Core Domain 2
GO: Gene Ontology
STRING: Search Tool for the Retrieval of Interacting Genes/Proteins
TLRs: Toll-like receptors
TNNI3: Troponin I
CALCA: Calcitonin
