# Supplementary material for: Persistence of dysfunctional immune response 12 months after SARS-CoV-2 infection and their relationship with pulmonary sequelae and long COVID
Source: Respir Res. 2025 Apr 17;26:152. doi: 10.1186/s12931-025-03200-1 (PMC12004571; doi:10.1186/s12931-025-03200-1)
Supplement: Supplementary file 1 — Supplementary Material 1. [file 12931_2025_3200_MOESM1_ESM.docx]

**Persistence of dysfunctional immune response 12 months after SARS-CoV-2 infection and their relationship with pulmonary sequelae and long covid.**

Tamara Cruz^1,2^, Núria Albacar^1, 2,3^, Estibaliz Ruiz^1,4^, Gema M Lledo^5^, Lídia Perea^1,2^, Alba Puebla^1,6^, Alejandro Torvisco^1,6^, Núria Mendoza^1^, Pau Marrades^3^, Jacobo Sellares^1,2,3^, Alvar Agustí^1,2,3^, Odette Viñas^1,4^, Oriol Sibila^1,2,3*^, Rosa Faner^1,2,6 *^

**SUPPLEMENTARY FIGURES:**

**Supplementary figure 1.** Accumulation of autoantibodies in Post-COVID patients. The dot-plots show the number of autoreactivies per patient by group, of anti-nuclear, anti-cytoplasmic, anti-inf-g, or the total number of positivity. Rec= recovered; PS= Pulmonary Sequelae and LC= long COVID.

**
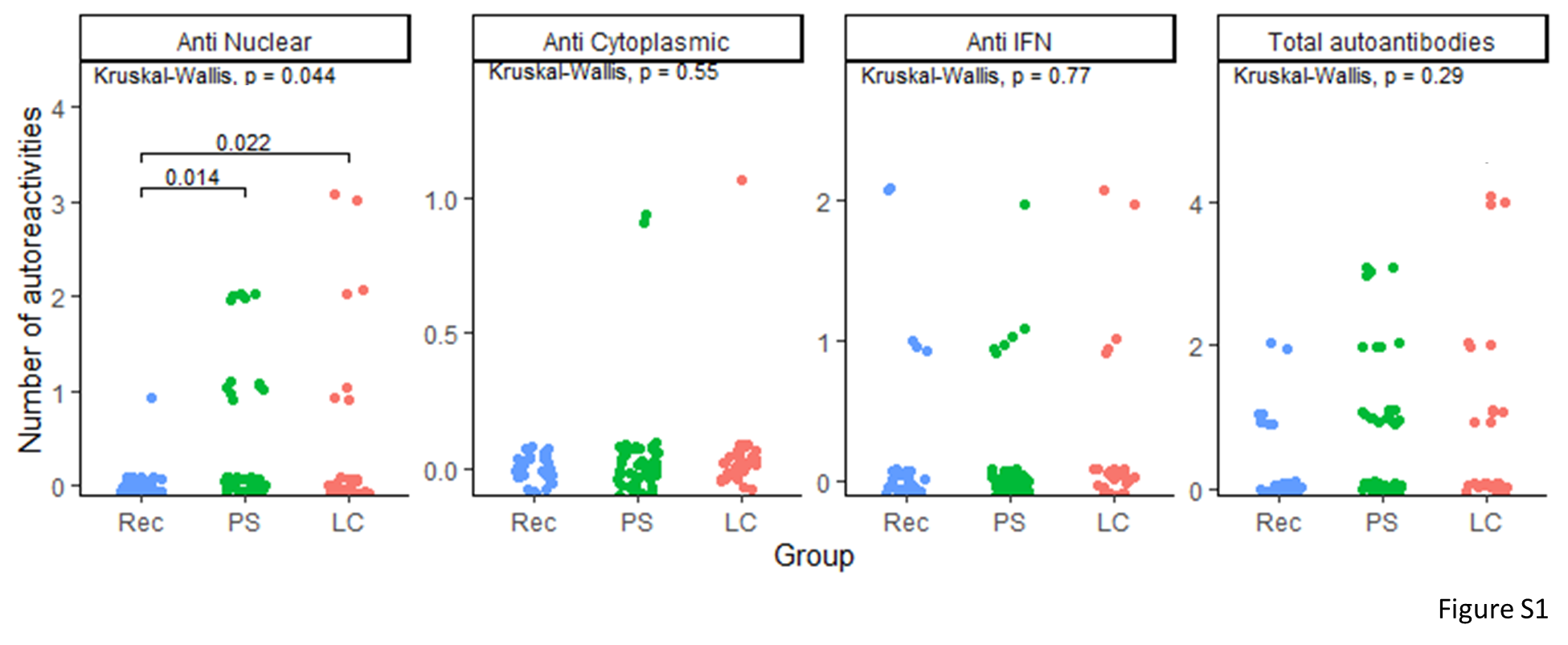
**

**Supplementary figure 2.** String protein-protein interaction networks of the inflammation proteins with increase expression in the following comparison. A) PS vs recovered, B) LC vs recovered, C) PS vs LC and D) LC vs PS. **
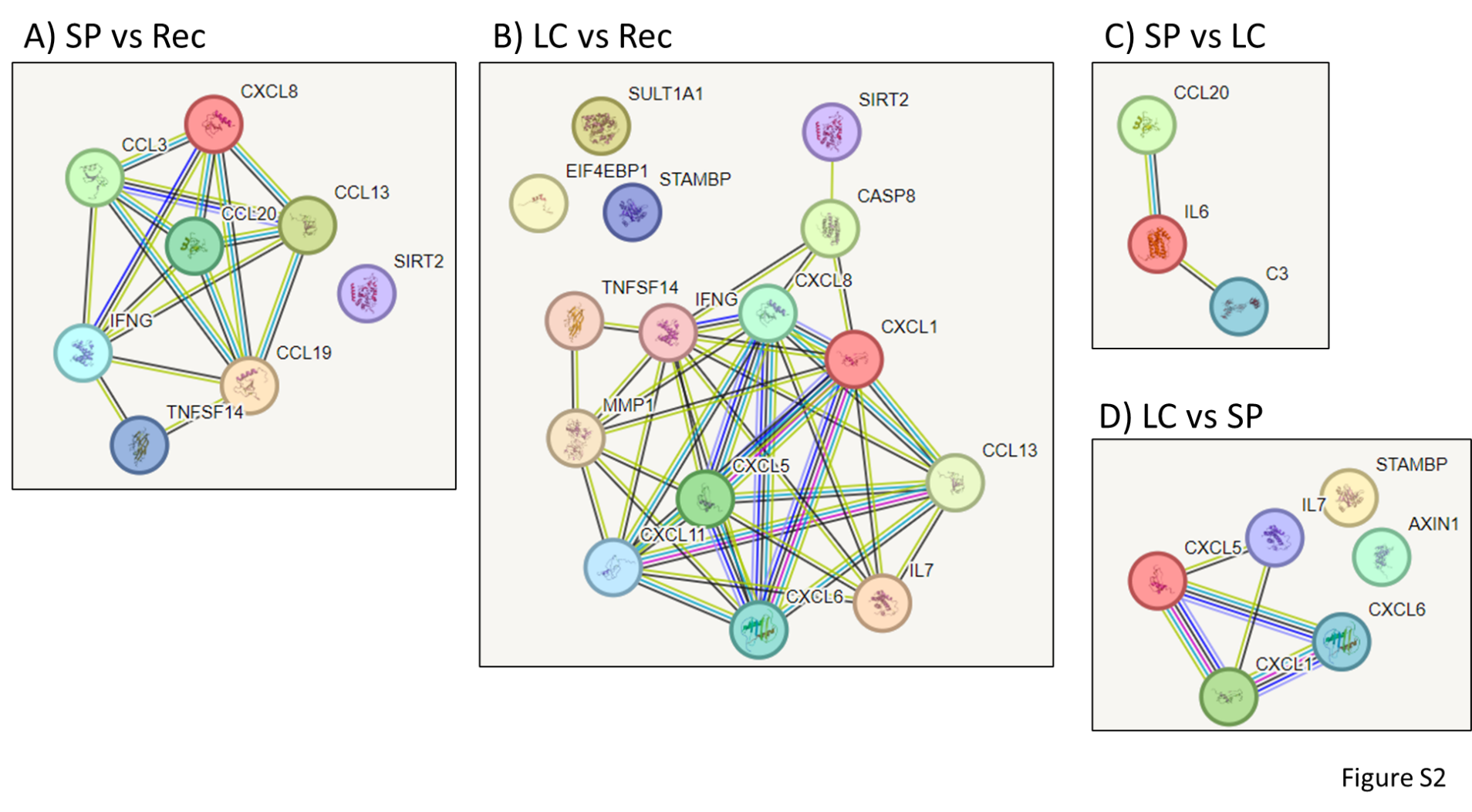
**

**SUPPLEMENTARY TABLES:**

**Supplementary table 1.** Plasma inflammatory markers comparing PS, LC and the recovered. Results are express as mean and standard deviation or the 95% confidence interquartile and the statistical test was performed using a Kruskal-Wallis with post-hoc FDR correction.

**Supplementary table 2.** Plasma inflammatory proteins in PS correlation with disease severity and evolution from 6 to 12 months of convalescence

**Supplementary table 3.** Biological functions of the plasma proteins over-express in PS and LC patients in comparison with the recovered.

**Supplementary table 4.** Biological functions of the plasma proteins over-express in the comparison between PS and LC patients.

**Supplementary table 5.** Plasma levels of organ-damage relate proteins comparing PS, LC and the recovered. Results are express as mean and standard deviation or the 95% confidence interquartile and the statistical test was performed using a Kruskal-Wallis with post-hoc FDR correction.

**Supplementary table 6.** Main functions of the organ-damage relate proteins with altered levels in the LC group.

**Supplementary table 1.** Plasma inflammatory markers comparing PS, LC and the recovered. Results are express as mean and standard deviation or the 95% confidence interquartile and the statistical test was performed using a Kruskal-Wallis with post-hoc FDR correction.

|  | Rec | PS | LC | Kruskal-Wallis | | | |
| --- | --- | --- | --- | --- | --- | --- | --- |
|  | N=31 | N=51 | N=31 | p-val | p-val Rec vs PS | p-val Rec vs LC | p-val PS vs LC |
| C3 | 1.11 (0.20) | 1.21 (0.20) | 1.10 (0.19) | **0.023** | **0.065** | 0.910 | **0.037** |
| STAMBP | 6.37 (1.48) | 6.81 (1.32) | 7.42 (0.92) | **0.003** | **0.081** | **0.001** | **0.032** |
| SIRT2 | 6.81 [5.21;7.19] | 7.42 [6.32;8.10] | 7.85 [7.28;8.44] | **0.003** | **0.041** | **0.001** | **0.053** |
| TNFSF14 | 5.28 (1.14) | 5.81 (0.99) | 6.04 (0.83) | **0.003** | **0.011** | **0.001** | **0.271** |
| CXCL1 | 9.57 [8.45;10.1] | 9.75 [8.93;10.2] | 10.3 [9.73;10.5] | **0.005** | 0.596 | **0.002** | 0.010 |
| CXCL6 | 8.83 (1.27) | 9.10 (1.16) | 9.67 (0.87) | **0.006** | 0.205 | **0.002** | **0.033** |
| CXCL5 | 10.9 [10.0;11.7] | 11.2 [10.2;11.8] | 12.2 [11.7;12.5] | **0.002** | 0.5 | **0.004** | 0.004 |
| 4E-BP1 | 9.17 (1.08) | 9.54 (0.99) | 9.83 (0.70) | **0.031** | 0.145 | **0.005** | **0.221** |
| MCP-4 | 13.5 (0.97) | 13.9 (0.90) | 14.0 (0.77) | **0.022** | **0.03** | **0.006** | **0.723** |
| CASP-8 | 2.74 [2.49;3.28] | 3.14 [2.54;3.89] | 3.54 [3.10;4.09] | **0.035** | 0.114 | **0.008** | 0.251 |
| IL7 | 2.59 (1.02) | 2.75 (0.93) | 3.15 (0.78) | **0.018** | 0.253 | **0.01** | **0.045** |
| ST1A1 | 3.64 [2.96;4.20] | 4.07 [3.19;4.40] | 4.38 [3.79;4.60] | **0.025** | 0.232 | **0.012** | 0.075 |
| MMP-1 | 13.3 (1.13) | 13.7 (1.20) | 14.0 (1.03) | **0.053** | 0.137 | **0.013** | **0.280** |
| IFN-gamma | 5.36 [4.67;6.06] | 5.86 [5.39;6.41] | 5.90 [5.56;6.47] | **0.030** | **0.028** | **0.014** | 0.695 |
| IL8 | 3.77 [3.48;4.19] | 4.49 [3.64;5.14] | 4.27 [3.96;4.68] | **0.034** | **0.028** | **0.015** | **0.878** |
| CXCL11 | 7.94 (1.04) | 8.28 (1.06) | 8.56 (0.90) | 0.100 | 0.153 | **0.032** | 0.379 |
| CCL3 | 6.02 [5.73;6.46] | 6.70 [6.03;7.03] | 6.39 [6.06;6.63] | **0.009** | **0.003** | **0.05** | 0.284 |
| CCL20 | 7.36 [6.90;7.76] | 7.77 [7.25;8.16] | 6.98 [6.77;7.49] | **0.002** | **0.04** | 0.179 | 0.003 |
| CCL19 | 8.52 [8.00;8.83] | 8.86 [8.29;9.72] | 8.59 [8.34;8.97] | **0.049** | **0.02** | 0.188 | 0.200 |
| IL6 | 3.02 [2.75;3.38] | 3.31 [2.85;3.66] | 2.87 [2.59;3.20] | **0.025** | 0.122 | 0.245 | 0.029 |
| FGF-19 | 8.54 (0.71) | 8.44 (1.03) | 8.41 (0.93) | 0.798 | 0.95 | 0.342 | 0.954 |
| OSM | 3.76 [3.41;4.30] | 3.95 [3.43;4.93] | 4.12 [3.53;4.83] | 0.560 | 0.337 | 0.349 | 0.909 |
| C4 | 0.27 (0.09) | 0.31 (0.09) | 0.30 (0.09) | 0.173 | 0.189 | 0.381 | 0.566 |
| EN-RAGE | 2.38 [2.05;2.59] | 2.34 [2.09;2.90] | 2.25 [2.03;2.72] | 0.709 | 0.778 | 0.569 | 0.782 |
| FGF-21 | 5.21 (1.25) | 5.60 (1.33) | 5.40 (1.36) | 0.624 | 0.351 | 0.627 | 0.634 |
| CXCL10 | 8.60 [8.08;9.42] | 8.91 [8.43;9.69] | 8.67 [8.44;9.09] | 0.246 | 0.14 | 0.688 | 0.321 |
| MCP-2 | 9.38 (0.98) | 9.57 (0.71) | 9.35 (0.82) | 0.201 | 0.083 | 0.773 | 0.371 |
| IL-17C | 2.54 [2.34;2.91] | 2.62 [2.29;2.96] | 2.58 [2.21;3.04] | 0.902 | 0.713 | 0.916 | 0.922 |
| CXCL9 | 6.69 [6.13;7.20] | 7.01 [6.25;7.60] | 6.61 [6.31;7.03] | 0.233 | 0.202 | 0.983 | 0.305 |

**Supplementary table 2.** **Plasma inflammatory proteins in PS correlation with disease severity and evolution from 6 to 12 months of convalescence.** The 8 markers with elevated levels at 12 months in PS were compared also at 6 months of convalescence. Their change was analyzed with a pair t-test and their association with severity of the pulmonary impairment by correlation with the DLCO. Results are express as mean and standard deviation or the 95% confidence interquartile and the statistical test was performed using a Mann-Whitney, the pair t-test represents de average difference (dif.) between 6 and 12 months and correlations were performed using Spearman.

|  | 12 months | | | 6 months | | | Pair t-test 6 to 12 months | | DLCO at 12m | | DLCO change 6 to 12 | |
| --- | --- | --- | --- | --- | --- | --- | --- | --- | --- | --- | --- | --- |
|  | Rec (n=31) | PS (n=51) | p-val | Rec (n=31) | PS (n=51) | p-val | dif. | p-val | r | p-val | r | p-val |
| CCL3 | 6.02 [5.73;6.46] | 6.70 [6.03;7.03] | **0.003** | 6.15 (0.61) | 6.33 (0.47) | 0.146 | -0.242 | **0.006** | -0.3276 | **0.0000** | 0.1092 | 0.1721 |
| CCL19 | 8.52 [8.00;8.83] | 8.86 [8.29;9.72] | **0.02** | 8.76 [8.31;8.99] | 8.77 [8.48;9.27] | 0.273 | -0.184 | **0.017** | -0.3068 | **0.0001** | 0.0772 | 0.3349 |
| CCL20 | 7.36 [6.90;7.76] | 7.77 [7.25;8.16] | **0.04** | 7.51 [7.03;7.81] | 7.77 [7.35;8.47] | **0.035** | 0.204 | 0.277 | -0.2555 | **0.0012** | 0.1678 | **0.0351** |
| IFN-gamma | 5.36 [4.67;6.06] | 5.86 [5.39;6.41] | **0.028** | 5.29 [4.99;5.97] | 5.89 [5.27;6.44] | **0.025** | -0.100 | 0.599 | -0.2920 | **0.0002** | 0.0585 | 0.4650 |
| IL8 | 3.77 [3.48;4.19] | 4.49 [3.64;5.14] | **0.028** | 3.84 [3.59;4.63] | 4.27 [3.73;4.77] | 0.16 | -0.128 | 0.339 | -0.2569 | **0.0011** | -0.0343 | 0.6692 |
| MCP-4 | 13.5 (0.97) | 13.9 (0.90) | **0.03** | 13.4 [12.5;13.9] | 13.5 [12.8;14.1] | 0.375 | -0.293 | 0.059 | -0.1679 | **0.0349** | 0.0598 | 0.4552 |
| TNFSF14 | 5.28 (1.14) | 5.81 (0.99) | **0.011** | 5.44 (1.18) | 5.37 (0.80) | 0.774 | -0.307 | 0.060 | -0.0781 | 0.3295 | -0.1097 | 0.1699 |
| SIRT2 | 6.81 [5.21;7.19] | 7.42 [6.32;8.10] | **0.041** | 6.80 (1.74) | 6.67 (1.48) | 0.709 | -0.342 | 0.289 | 0.0181 | 0.8216 | -0.0610 | 0.4462 |

**Supplementary table 3.** Biological functions of the plasma proteins over-express in PS and LC patients in comparison with the recovered.

|  |  |  | PS Vs Rec | | | LC Vs Rec | | |
| --- | --- | --- | --- | --- | --- | --- | --- | --- |
| GO | Description | GO genes | Proteins | Strength | FDR | Proteins | Strength | FDR |
| Altered biology process in both PS and LC in comparison with the recovered group | | | | | | | | |
| GO:0002274 | Myeloid leukocyte activation | 150 | 3 | 1.69 | 0.0039 | 3 | 1.42 | 0.0456 |
| GO:0010818 | T cell chemotaxis | 16 | 2 | 2.49 | 0.0036 | 2 | 2.22 | 0.0204 |
| GO:0048247 | Lymphocyte chemotaxis | 51 | 5 | 2.38 | 9.31E-08 | 3 | 1.89 | 0.0026 |
| GO:0030593 | Neutrophil chemotaxis | 80 | 5 | 2.19 | 2.88E-07 | 6 | 1.99 | 1.46E-07 |
| GO:0070098 | Chemokine-mediated signaling pathway | 82 | 5 | 2.18 | 2.88E-07 | 6 | 1.98 | 1.46E-07 |
| GO:0031640 | Killing of cells of another organism | 71 | 4 | 2.14 | 6.56E-06 | 4 | 1.87 | 0.00011 |
| GO:0030595 | Leukocyte chemotaxis | 149 | 6 | 2 | 9.31E-08 | 7 | 1.79 | 1.24E-07 |
| GO:0061844 | Antimicrobial humoral immune response mediated by antimicrobial peptide | 113 | 4 | 1.94 | 2.89E-05 | 6 | 1.84 | 2.67E-07 |
| GO:0006959 | Humoral immune response | 268 | 5 | 1.66 | 1.16E-05 | 8 | 1.59 | 1.24E-07 |
| GO:0019221 | Cytokine-mediated signaling pathway | 369 | 6 | 1.6 | 9.70E-07 | 8 | 1.45 | 1.66E-07 |
| GO:0071219 | Cellular response to molecule of bacterial origin | 205 | 3 | 1.56 | 0.0084 | 6 | 1.58 | 5.34E-06 |
| GO:0006954 | Inflammatory response | 538 | 6 | 1.44 | 6.15E-06 | 7 | 1.23 | 3.49E-05 |
| GO:0045321 | Leukocyte activation | 574 | 5 | 1.33 | 0.00026 | 6 | 1.14 | 0.00084 |
| GO:0098542 | Defense response to other organism | 989 | 7 | 1.24 | 4.04E-06 | 8 | 1.03 | 8.99E-05 |
| GO:0032101 | Regulation of response to external stimulus | 964 | 6 | 1.19 | 9.91E-05 | 6 | 0.91 | 0.0129 |
| GO:0006955 | Immune response | 1321 | 8 | 1.17 | 4.39E-07 | 10 | 1 | 3.64E-06 |
| GO:0007166 | Cell surface receptor signaling pathway | 2040 | 7 | 0.93 | 0.00023 | 11 | 0.85 | 9.92E-06 |
| GO:0071310 | Cellular response to organic substance | 2019 | 7 | 0.93 | 0.00021 | 10 | 0.81 | 0.00011 |
| GO:0009605 | Response to external stimulus | 2355 | 8 | 0.92 | 1.79E-05 | 10 | 0.75 | 0.00038 |
| GO:0070887 | Cellular response to chemical stimulus | 2609 | 8 | 0.88 | 3.27E-05 | 12 | 0.78 | 7.20E-06 |
| GO:0007165 | Signal transduction | 4714 | 8 | 0.62 | 0.0019 | 13 | 0.56 | 0.00021 |
| GO:0048522 | Positive regulation of cellular process | 5584 | 8 | 0.55 | 0.0059 | 12 | 0.45 | 0.0132 |
| Altered biology process in PS in comparison with the recovered group | | | | | | | | |
| GO:0048245 | Eosinophil chemotaxis | 17 | 2 | 2.46 | 0.0039 |  |  |  |
| GO:0140131 | Positive regulation of lymphocyte chemotaxis | 22 | 2 | 2.35 | 0.0056 |  |  |  |
| GO:0072678 | T cell migration | 35 | 3 | 2.32 | 9.95E-05 |  |  |  |
| GO:0090023 | Positive regulation of neutrophil chemotaxis | 25 | 2 | 2.29 | 0.007 |  |  |  |
| GO:2000403 | Positive regulation of lymphocyte migration | 43 | 3 | 2.24 | 0.00016 |  |  |  |
| GO:0036230 | Granulocyte activation | 33 | 2 | 2.17 | 0.0105 |  |  |  |
| GO:0043372 | Positive regulation of CD4-positive, alpha-beta T cell differentiation | 33 | 2 | 2.17 | 0.0105 |  |  |  |
| GO:2000406 | Positive regulation of T cell migration | 35 | 2 | 2.15 | 0.0114 |  |  |  |
| GO:0071347 | Cellular response to interleukin-1 | 98 | 5 | 2.1 | 2.88E-07 |  |  |  |
| GO:0032731 | Positive regulation of interleukin-1 beta production | 61 | 3 | 2.08 | 0.0004 |  |  |  |
| GO:0071346 | Cellular response to interferon-gamma | 105 | 5 | 2.07 | 3.06E-07 |  |  |  |
| GO:0031295 | T cell costimulation | 42 | 2 | 2.07 | 0.0148 |  |  |  |
| GO:0032735 | Positive regulation of interleukin-12 production | 43 | 2 | 2.06 | 0.0154 |  |  |  |
| GO:0002690 | Positive regulation of leukocyte chemotaxis | 99 | 4 | 2 | 2.00E-05 |  |  |  |
| GO:0051209 | Release of sequestered calcium ion into cytosol | 52 | 2 | 1.98 | 0.0209 |  |  |  |
| GO:0043491 | Protein kinase B signaling | 53 | 2 | 1.97 | 0.0214 |  |  |  |
| GO:0043388 | Positive regulation of DNA binding | 58 | 2 | 1.93 | 0.024 |  |  |  |
| GO:0002687 | Positive regulation of leukocyte migration | 151 | 5 | 1.91 | 1.20E-06 |  |  |  |
| GO:0002275 | Myeloid cell activation involved in immune response | 61 | 2 | 1.91 | 0.0258 |  |  |  |
| GO:1901224 | Positive regulation of NIK/NF-kappaB signaling | 67 | 2 | 1.87 | 0.0306 |  |  |  |
| GO:0032760 | Positive regulation of tumor necrosis factor production | 101 | 3 | 1.86 | 0.0014 |  |  |  |
| GO:0071356 | Cellular response to tumor necrosis factor | 175 | 5 | 1.85 | 2.25E-06 |  |  |  |
| GO:0045670 | Regulation of osteoclast differentiation | 70 | 2 | 1.85 | 0.0329 |  |  |  |
| GO:0098586 | Cellular response to virus | 87 | 2 | 1.75 | 0.0475 |  |  |  |
| GO:0070374 | Positive regulation of ERK1 and ERK2 cascade | 213 | 4 | 1.67 | 0.00023 |  |  |  |
| GO:0019722 | Calcium-mediated signaling | 169 | 3 | 1.64 | 0.0052 |  |  |  |
| GO:0006874 | Cellular calcium ion homeostasis | 193 | 3 | 1.58 | 0.0072 |  |  |  |
| GO:0043547 | Positive regulation of GTPase activity | 285 | 4 | 1.54 | 0.00062 |  |  |  |
| GO:0042063 | Gliogenesis | 248 | 3 | 1.47 | 0.0126 |  |  |  |
| GO:0050870 | Positive regulation of T cell activation | 251 | 3 | 1.47 | 0.0129 |  |  |  |
| GO:0030335 | Positive regulation of cell migration | 529 | 6 | 1.45 | 5.83E-06 |  |  |  |
| GO:0032103 | Positive regulation of response to external stimulus | 453 | 5 | 1.43 | 9.82E-05 |  |  |  |
| GO:0002696 | Positive regulation of leukocyte activation | 384 | 4 | 1.41 | 0.0017 |  |  |  |
| GO:1902105 | Regulation of leukocyte differentiation | 315 | 3 | 1.37 | 0.0216 |  |  |  |
| GO:0051345 | Positive regulation of hydrolase activity | 589 | 5 | 1.32 | 0.00029 |  |  |  |
| GO:0045087 | Innate immune response | 754 | 6 | 1.29 | 2.95E-05 |  |  |  |
| GO:0002684 | Positive regulation of immune system process | 874 | 6 | 1.23 | 6.22E-05 |  |  |  |
| GO:0009617 | Response to bacterium | 663 | 4 | 1.17 | 0.0104 |  |  |  |
| GO:0051336 | Regulation of hydrolase activity | 1011 | 6 | 1.16 | 0.00012 |  |  |  |
| GO:0030155 | Regulation of cell adhesion | 784 | 4 | 1.1 | 0.0165 |  |  |  |
| GO:1902533 | Positive regulation of intracellular signal transduction | 997 | 5 | 1.09 | 0.0027 |  |  |  |
| GO:0045597 | Positive regulation of cell differentiation | 876 | 4 | 1.05 | 0.0229 |  |  |  |
| GO:0010629 | Negative regulation of gene expression | 899 | 4 | 1.04 | 0.0248 |  |  |  |
| GO:0007186 | G protein-coupled receptor signaling pathway | 1174 | 5 | 1.02 | 0.0053 |  |  |  |
| GO:0044093 | Positive regulation of molecular function | 1587 | 6 | 0.97 | 0.0013 |  |  |  |
| GO:0051094 | Positive regulation of developmental process | 1332 | 5 | 0.97 | 0.0089 |  |  |  |
| GO:0051130 | Positive regulation of cellular component organization | 1049 | 4 | 0.97 | 0.0416 |  |  |  |
| GO:2000026 | Regulation of multicellular organismal development | 1389 | 5 | 0.95 | 0.0104 |  |  |  |
| GO:0042981 | Regulation of apoptotic process | 1462 | 5 | 0.93 | 0.0122 |  |  |  |
| GO:0048584 | Positive regulation of response to stimulus | 2131 | 7 | 0.91 | 0.00029 |  |  |  |
| GO:0045595 | Regulation of cell differentiation | 1582 | 5 | 0.89 | 0.0163 |  |  |  |
| GO:0050793 | Regulation of developmental process | 2492 | 7 | 0.84 | 0.00073 |  |  |  |
| GO:0009966 | Regulation of signal transduction | 2978 | 7 | 0.76 | 0.0022 |  |  |  |
| GO:0065009 | Regulation of molecular function | 3085 | 7 | 0.75 | 0.0027 |  |  |  |
| GO:0048583 | Regulation of response to stimulus | 3931 | 8 | 0.7 | 0.00055 |  |  |  |
| Altered biology process in LC in comparison with the recovered group | | | | | | | | |
| GO:0002237 | Response to molecule of bacterial origin | 333 |  |  |  | 3 | 1.61 | 0.014 |
| GO:0002376 | Immune system process | 2121 |  |  |  | 3 | 1.54 | 0.021 |
| GO:0032496 | Response to lipopolysaccharide | 314 |  |  |  | 7 | 1.44 | 2.25E-06 |
| GO:0032642 | Regulation of chemokine production | 96 |  |  |  | 6 | 1.4 | 4.04E-05 |
| GO:0034097 | Response to cytokine | 804 |  |  |  | 9 | 1.17 | 1.30E-06 |
| GO:0042127 | Regulation of cell population proliferation | 1669 |  |  |  | 9 | 0.95 | 5.15E-05 |
| GO:0051707 | Response to other organism | 1328 |  |  |  | 6 | 0.87 | 0.0201 |
| GO:0051716 | Cellular response to stimulus | 6357 |  |  |  | 11 | 0.83 | 1.36E-05 |
| GO:0097191 | Extrinsic apoptotic signaling pathway | 113 |  |  |  | 8 | 0.8 | 0.0029 |
| GO:1901700 | Response to oxygen-containing compound | 1547 |  |  |  | 7 | 0.77 | 0.0175 |
| GO:1901701 | Cellular response to oxygen-containing compound | 1057 |  |  |  | 15 | 0.49 | 2.72E-05 |

**Supplementary table 4.** Biological functions of the plasma proteins over-express in the comparison between PS and LC patients.

| GO | Desription | Gen number | Proteins | Strength | FDR |
| --- | --- | --- | --- | --- | --- |
| Biology process overexpress in PS in comparison with the LC | | |  |  |  |
| GO:0002675 | Positive regulation of acute inflammatory response | 27 | 2 | 2.69 | 0.0492 |
| GO:0010575 | Positive regulation of vascular endothelial growth factor production | 29 | 2 | 2.66 | 0.0492 |
| GO:0002548 | Monocyte chemotaxis | 43 | 2 | 2.48 | 0.0492 |
| GO:0006959 | Humoral immune response | 268 | 3 | 1.87 | 0.0404 |
| Biology process overexpress in LC in comparison with the PS | | |  |  |  |
| GO:0030593 | Neutrophil chemotaxis | 80 | 3 | 2.09 | 0.0098 |
| GO:0070098 | Chemokine-mediated signaling pathway | 82 | 3 | 2.08 | 0.0098 |
| GO:0061844 | Antimicrobial humoral immune response mediated by antimicrobial peptide | 113 | 3 | 1.94 | 0.0098 |
| GO:0071222 | Cellular response to lipopolysaccharide | 195 | 3 | 1.7 | 0.0204 |
| GO:0006959 | Humoral immune response | 268 | 4 | 1.69 | 0.0082 |
| GO:0019221 | Cytokine-mediated signaling pathway | 369 | 4 | 1.55 | 0.0098 |
| GO:0007166 | Cell surface receptor signaling pathway | 2040 | 6 | 0.98 | 0.0098 |

**Supplementary table 5.** Plasma levels of organ-damage relate proteins comparing PS, LC and the recovered. Results are express as mean and standard deviation or the 95% confidence interquartile and the statistical test was performed using a Kruskal-Wallis with post-hoc FDR correction.

|  | Rec | PS | LC | Kruskal-Wallis | | | |
| --- | --- | --- | --- | --- | --- | --- | --- |
|  | N=31 | N=51 | N=31 | p-val | p-val Rec vs PS | p-val Rec vs LC | p-val PS vs LC |
| TNNI3 | 5.06 [3.89;5.96] | 4.51 [3.41;5.93] | 3.24 [1.60;4.17] | **0.002** | 0.426 | **0.002** | **0.008** |
| WAS | 3.54 [2.33;4.56] | 3.77 [2.71;4.77] | 4.94 [3.60;6.29] | **0.005** | 0.485 | **0.009** | **0.009** |
| FKBP1B | 2.03 (0.98) | 2.20 (0.88) | 2.68 (0.78) | **0.007** | 0.280 | **0.012** | **0.019** |
| STX8 | 3.93 (1.18) | 4.06 (0.91) | 4.69 (0.64) | **0.003** | 0.516 | **0.012** | **0.003** |
| PTPRJ | 3.08 (1.35) | 3.21 (1.16) | 3.86 (0.89) | **0.005** | 0.308 | **0.013** | **0.010** |
| MAX | 2.32 (1.45) | 2.51 (1.21) | 3.08 (0.90) | **0.010** | 0.259 | **0.017** | **0.028** |
| BANK1 | 8.76 [7.84;10.1] | 9.15 [8.63;9.87] | 9.75 [9.41;10.3] | **0.007** | 0.450 | **0.018** | **0.012** |
| FOXO1 | 4.71 (1.62) | 4.86 (1.38) | 5.54 (0.88) | **0.017** | 0.563 | **0.020** | **0.020** |
| MVK | 3.79 (1.29) | 4.00 (1.00) | 4.42 (0.81) | **0.013** | 0.214 | **0.026** | **0.033** |
| NUB1 | 3.37 [2.60;4.83] | 3.78 [3.11;4.67] | 4.46 [3.76;5.33] | **0.015** | 0.297 | **0.027** | **0.027** |
| YES1 | 7.57 [6.54;9.34] | 8.12 [7.03;8.69] | 8.67 [8.29;9.28] | **0.009** | 0.573 | **0.028** | **0.010** |
| ERBB2IP | 4.67 [3.79;5.90] | 5.03 [4.41;5.41] | 5.48 [5.16;5.98] | **0.009** | 0.582 | **0.030** | **0.009** |
| RRM2B | 2.85 (1.28) | 2.81 (1.02) | 3.31 (0.66) | **0.024** | 0.576 | **0.036** | **0.036** |
| AIFM1 | 3.54 (1.82) | 3.68 (1.48) | 4.53 (0.98) | **0.018** | 0.695 | **0.036** | **0.024** |
| TOP2B | 4.66 (1.86) | 4.73 (1.34) | 5.38 (1.02) | **0.019** | 0.462 | **0.041** | **0.027** |
| PRKAB1 | 3.08 (1.21) | 3.12 (1.01) | 3.59 (0.72) | **0.024** | 0.702 | **0.044** | **0.033** |
| CSNK1D | 1.31 [0.49;2.62] | 1.63 [1.20;2.21] | 1.86 [1.70;2.39] | **0.027** | 0.424 | **0.048** | **0.045** |
| SMAD1 | 3.51 (1.81) | 3.74 (1.53) | 4.46 (0.95) | **0.028** | 0.528 | 0.053 | **0.039** |
| LHB | 1.31 [0.97;1.97] | 1.79 [1.30;2.49] | 1.76 [1.32;2.79] | 0.058 | 0.089 | 0.058 | 0.731 |
| BID | 1.08 [0.69;2.33] | 1.52 [0.83;2.27] | 1.89 [1.29;2.81] | 0.055 | 0.202 | 0.063 | 0.193 |
| MAP4K5 | 8.14 [6.90;9.77] | 8.57 [8.00;9.17] | 9.19 [8.59;9.68] | **0.023** | 0.444 | 0.063 | **0.026** |
| VASH1 | 1.33 (1.81) | 1.68 (1.33) | 2.25 (0.89) | **0.038** | 0.327 | 0.063 | 0.063 |
| BTC | 2.27 [1.67;2.69] | 2.45 [2.02;3.02] | 2.71 [2.27;3.39] | **0.047** | 0.135 | 0.064 | 0.230 |
| NCF2 | 5.03 [4.64;6.64] | 5.78 [4.91;7.27] | 6.36 [5.61;8.05] | **0.048** | 0.225 | 0.071 | 0.123 |
| NUCB2 | 5.51 (1.27) | 5.68 (1.01) | 6.05 (0.76) | 0.061 | 0.389 | 0.072 | 0.072 |
| INPPL1 | 5.84 [4.66;7.37] | 6.21 [5.21;6.53] | 6.66 [6.32;7.08] | **0.015** | 0.702 | 0.088 | **0.008** |
| CALCA | 5.01 [4.65;5.47] | 5.14 [4.78;5.62] | 4.66 [4.40;5.04] | **0.007** | 0.228 | 0.097 | **0.006** |
| PVALB | 8.28 [6.67;9.78] | 9.03 [7.09;9.90] | 9.63 [8.69;10.1] | 0.088 | 0.586 | 0.104 | 0.104 |
| RASSF2 | 1.97 [1.64;2.86] | 2.01 [1.69;2.88] | 2.45 [2.04;3.37] | 0.116 | 0.928 | 0.123 | 0.123 |
| LAT2 | 5.92 (1.90) | 6.06 (1.43) | 6.72 (0.84) | 0.072 | 0.871 | 0.129 | 0.078 |
| NBN | 2.68 [2.26;4.81] | 3.32 [2.60;4.20] | 4.36 [3.22;5.39] | 0.069 | 0.560 | 0.133 | 0.076 |
| PXN | 2.83 [2.45;3.21] | 2.83 [2.46;3.62] | 3.41 [2.79;4.14] | 0.146 | 0.702 | 0.136 | 0.136 |
| TIGAR | 2.30 [1.81;3.00] | 2.64 [2.10;3.29] | 2.94 [2.37;3.52] | 0.136 | 0.306 | 0.140 | 0.306 |
| FGR | 1.97 [1.21;3.53] | 2.20 [1.72;2.92] | 2.87 [2.12;3.42] | 0.101 | 0.462 | 0.145 | 0.145 |
| PRKRA | 1.89 [1.52;2.65] | 2.26 [1.70;2.90] | 2.39 [1.89;3.13] | 0.173 | 0.341 | 0.188 | 0.341 |
| CRH | 2.20 [1.83;2.83] | 2.62 [2.06;3.15] | 2.65 [1.92;3.36] | 0.274 | 0.247 | 0.247 | 0.745 |
| TMPRSS15 | 1.72 (0.94) | 2.20 (1.12) | 2.07 (0.82) | 0.223 | 0.281 | 0.281 | 0.681 |
| SERPINA9 | 2.22 [0.95;2.57] | 2.15 [0.95;2.69] | 2.30 [2.15;2.64] | 0.222 | 0.741 | 0.366 | 0.258 |
| AGR2 | 3.94 [3.00;4.25] | 3.42 [2.98;4.20] | 4.07 [3.36;4.38] | 0.137 | 0.387 | 0.387 | 0.152 |
| NOS3 | 3.15 [2.70;3.64] | 3.28 [2.79;3.68] | 3.03 [2.74;3.22] | 0.127 | 0.456 | 0.456 | 0.114 |
| KIM1 | 7.90 [7.24;8.31] | 8.05 [7.54;8.83] | 7.55 [7.27;8.15] | **0.040** | 0.169 | 0.458 | **0.047** |
| PTN | 3.06 [2.28;3.73] | 3.09 [2.29;3.80] | 2.70 [2.22;3.07] | 0.371 | 0.863 | 0.522 | 0.471 |
| ADGRG1 | 1.20 [0.97;1.48] | 1.31 [1.06;2.38] | 1.16 [0.96;1.36] | 0.062 | 0.106 | 0.746 | 0.106 |
| CAPG | 2.88 [2.61;3.68] | 3.19 [2.27;3.87] | 3.37 [2.70;3.51] | 0.828 | 0.848 | 0.848 | 0.848 |
| NPPC | 3.56 [2.94;3.96] | 3.69 [3.27;4.09] | 3.48 [3.09;3.93] | 0.266 | 0.300 | 0.978 | 0.300 |
| FOSB | 0.51 [0.29;1.34] | 0.62 [0.30;0.99] | 0.69 [0.32;1.12] | 0.956 | 1.000 | 1.000 | 1.000 |

**Supplementary table 6.** Main functions of the proteins with altered levels in the LC group.

| **Protein** | **Full name** | **Function** | **References** |
| --- | --- | --- | --- |
| **COVID-association** | |  |  |
| TNNI3 | Troponin I3, cardiac type | Associated with severe COVID in the acute episode | ^1^ |
| CALCA | Calcitonin | Elevated serum levels associated with COVID19 disease severity and mortality | ^2^ |
| KIM1 | Hepatitis A Virus Cellular Receptor 1 | Possible entry factor for SARS-CoV-2 and other coronaviruses, may thus mediate and exacerbate the renal infection of SARS-CoV-2. Potential mitigator of COVID-19 severity | ^3,4^ |
| FKBP1B | Peptidyl-prolyl cis-trans isomerase FKBP1B | Potential target for replication of human coronaviruses | ^5^ |
| PRKAB1 | AMP-activated protein kinase subunit beta-1 | Modification of AMPK activity in benefit of viral replication | ^6^ |
| CSNK1D | Casein kinase I isoform delta | Phosphorilation of the viral N protein, which is required for viral replication | ^2^ |
| SMAD1 | Mothers against decapentaplegic homolog 1 | Increase endothelial permeabilization downstream BMPs. SMAD1 inhibition in mice model of SARS-CoV2 infection reduce mortality and severity. | ^7^ |
| FOXO1 | Forkhead box protein O1 | Promotes airway inflammation. Knock-out mice model protected from severe COVID19 inflammation disease. | ^8^ |
| MAP4K5 | Mitogen-activated protein kinase kinase kinase kinase 5 | Inactivated by the main viral protease of SARS-CoV2 (3CLpro) | ^9^ |
| MAX | Protein max | SARS_CoV2 blocks the antagonistic MGA/MAX complex, which shifts the balance towards MYC/MAX and activates glycolysis | ^5^ |
| AIFM1 | Apoptosis-inducing factor 1, mitochondrial | Molecular mimicry with SARS-CoV-2 | ^10^ |
| STX8 | Syntaxin-8 | Increased in pleural samples of deceased COVID-19 patients | ^11^ |
| **Inflammation/immune related** | |  |  |
| KIM1 | Hepatitis A Virus Cellular Receptor 1 | T-cell trafficking during inflammatory responses and the induction of autoimmune disease. Moderation of asthma and allergic diseases | ^12^ |
| MVK | Mevalonate kinase | Deficiency cause a Systemic autoinflammatory diseases (SAID) | ^13^ |
| BANK1 | B-Cell Scaffold Protein With Ankyrin Repeats | TLR7 and TLR9 B-cell activation, cytokine production and IgG production. Implicated in autoinmune diseases | ^14^ |
| STX8 | Syntaxin-8 | Vesicle trafficking protein is necessary for lytic granule trafficking in cytotoxic T lymphocytes | ^15^ |
| YES1 | Non-receptor Tyrosine-protein kinase Yes | Upon T-cell stimulation, induces T-cell migration | ^16^ |
| MAP4K5 | Mitogen-activated protein kinase kinase kinase kinase 5 | Immune cell signaling, immune responses, and inflammation. | ^17^ |
| RRM2B | Ribonucleoside-diphosphate reductase subunit M2 B | Critical role of RRM2B in mitochondrial homeostasis and the inflammation signaling pathway in a p53-independent manner under oxidative stress. | ^18^ |
| FOXO1 | Forkhead box protein O1 | Enhance inflammation in response to high glucose levels, TNF and LPS stimulation | ^19^ |
| ERBB2IP/ERBIN | Erbin | Inhibits NOD2-dependent NF-kappa-B signaling and proinflammatory cytokine secretion | ^20^ |
| INPPL1 | Inositol polyphosphate-5 phosphatase-like 1 | Regulation of neutrophil chemotaxis/trafficking and may promote inflammation | ^21^ |
| WAS | Wiskott-Aldrich syndrome protein | Expressed exclusively in hematopoietic cells, regulates the immune synapse formation, cell signaling, migration and cytokine release. | ^22^ |
| PTPRJ | Receptor-type tyrosine-protein phosphatase eta | Present in all hematopoietic lineages. Regulator of macrophage adhesion and spreading. After TCR activation down-regulate prolongation of signaling in T-cells. | ^23^ |
| SMAD1 | Mothers against decapentaplegic homolog 1 | Cell growth, apoptosis, morphogenesis, development and immune responses downstream bone morphogenetic proteins (BMPs) | ^24^ |
| **Cardiac related** | |  |  |
| TNNI3 | Troponin I3, cardiac type | Exclusively expressed in adult cardiac muscle | ^1,25^ |
| FKBP1B | Peptidyl-prolyl cis-trans isomerase FKBP1B | Excitation-contraction coupling in cardiac muscle | ^26^ |
| FOXO1 | Forkhead box protein O1 | [Mediates the function of MLIP in cardiomyocytes hypertrophy and cardiac remodeling](https://en.wikipedia.org/wiki/Adipogenesis) | ^27^ |
| **Stress response/environment sensor** | |  |  |
| MAX | Protein max | Governs many aspects of cell behavior, including cell proliferation, apoptosis and tumorigenesis. | ^28^ |
| FM1 | Apoptosis-inducing factor 1, mitochondrial | Caspase-independent apoptosis and regulation of respiratory chain biogenesis. | ^29^ |
| TOP2B | DNA topoisomerase 2-beta | Chromosome condensation, chromatid separation, and the relief of torsional stress that occurs during DNA transcription and replication | ^30^ |
| PRKAB1 | AMP-activated protein kinase subunit beta-1 | In response to reduction of intracellular ATP levels, AMPK activates energy-producing pathways and inhibits energy-consuming processes: inhibits protein, carbohydrate and lipid biosynthesis, as well as cell growth and proliferation. | ^31^ |
| CSNK1D | Casein kinase I isoform delta | Central component of the circadian clock, cell cycle, cellular growth and survival processes, DNA damage and cellular stress. | ^32^ |
| NUB1 | NEDD8 ultimate buster 1 | IFN inducible protein, involve in cell growth inhibition and anti-cancer | ^33^ |
| CALCA | Calcitonin | Inhibit bone reabsorption and enhance calcium excretion by the kidneys. | ^34^ |
| FOXO1 | Forkhead box protein O1 | [Negatively regulates adipogenesis, promotes apoptosis and mediate cell cycle arrest linked with cyclin D1 and cyclin D2. Mediates the function of MLIP in cardiomyocytes hypertrophy and cardiac remodeling](https://en.wikipedia.org/wiki/Adipogenesis) | ^19^ |
| SMAD1 | Mothers against decapentaplegic homolog 1 | Cell growth, apoptosis, morphogenesis and development | ^24^ |
| PTPRJ | Receptor-type tyrosine-protein phosphatase eta | Cell adhesion, migration, proliferation and differentiation. Involved in vascular development, platelet activation and thrombosis, enhances the barrier function of epithelial junctions during reassembly. | ^35^ |
| YES1 | Non-receptor Tyrosine-protein kinase Yes | Regulation of cell growth and survival, apoptosis, cell-cell adhesion, cytoskeleton remodeling, and differentiation. | ^36^ |
| RRM2B | Ribonucleoside-diphosphate reductase subunit M2 B | Cell survival by repairing damaged DNA in a p53-dependent manner, expression induced by p53 | ^18^ |

**References**

1. Duerr GD, Heine A, Hamiko M, et al. Parameters predicting COVID-19-induced myocardial injury and mortality. *Life Sci*. Sep 2020;260:118400. doi:10.1016/j.lfs.2020.118400

2. Ahmed S, Ahmed ZA, Rashid NH, Mansoor M, Siddiqui I, Jafri L. Procalcitonin as a predictor of severity and mortality in a cohort of patients hospitalised with COVID-19. *Malays J Pathol*. Dec 2021;43(3):375-380.

3. Vogel MJ, Mustroph J, Staudner ST, et al. Kidney injury molecule-1: potential biomarker of acute kidney injury and disease severity in patients with COVID-19. *J Nephrol*. Aug 2021;34(4):1007-1018. doi:10.1007/s40620-021-01079-x

4. Katopodis P, Randeva HS, Spandidos DA, Saravi S, Kyrou I, Karteris E. Host cell entry mediators implicated in the cellular tropism of SARS‑CoV‑2, the pathophysiology of COVID‑19 and the identification of microRNAs that can modulate the expression of these mediators (Review). *Int J Mol Med*. Feb 2022;49(2)doi:10.3892/ijmm.2021.5075

5. Zhu JY, Wang G, Huang X, et al. SARS-CoV-2 Nsp6 damages Drosophila heart and mouse cardiomyocytes through MGA/MAX complex-mediated increased glycolysis. *Commun Biol*. Sep 30 2022;5(1):1039. doi:10.1038/s42003-022-03986-6

6. Yaron TM, Heaton BE, Levy TM, et al. Host protein kinases required for SARS-CoV-2 nucleocapsid phosphorylation and viral replication. *Sci Signal*. Oct 25 2022;15(757):eabm0808. doi:10.1126/scisignal.abm0808

7. Morita M, Yoneda A, Tokunoh N, et al. Upregulation of Robo4 expression by SMAD signaling suppresses vascular permeability and mortality in endotoxemia and COVID-19 models. *Proc Natl Acad Sci U S A*. Jan 17 2023;120(3):e2213317120. doi:10.1073/pnas.2213317120

8. Sadhu S, Dalal R, Dandotiya J, et al. IL-9 aggravates SARS-CoV-2 infection and exacerbates associated airway inflammation. *Nat Commun*. Jul 10 2023;14(1):4060. doi:10.1038/s41467-023-39815-5

9. Razaghi A, Szakos A, Alouda M, Bozóky B, Björnstedt M, Szekely L. Proteomic Analysis of Pleural Effusions from COVID-19 Deceased Patients: Enhanced Inflammatory Markers. *Diagnostics (Basel)*. Nov 14 2022;12(11)doi:10.3390/diagnostics12112789

10. Bhutta MS, Gallo ES, Borenstein R. Multifaceted Role of AMPK in Viral Infections. *Cells*. May 06 2021;10(5)doi:10.3390/cells10051118

11. Sood T, Perrot N, Chong M, et al. Biomarkers Associated With Severe COVID-19 Among Populations With High Cardiometabolic Risk: A 2-Sample Mendelian Randomization Study. *JAMA Netw Open*. Jul 03 2023;6(7):e2325914. doi:10.1001/jamanetworkopen.2023.25914

12. Yi T, Song SU. Immunomodulatory properties of mesenchymal stem cells and their therapeutic applications. *Arch Pharm Res*. Feb 2012;35(2):213-21. doi:10.1007/s12272-012-0202-z

13. Gattorno M, Hofer M, Federici S, et al. Classification criteria for autoinflammatory recurrent fevers. *Ann Rheum Dis*. Aug 2019;78(8):1025-1032. doi:10.1136/annrheumdis-2019-215048

14. Gómez Hernández G, Morell M, Alarcón-Riquelme ME. The Role of BANK1 in B Cell Signaling and Disease. *Cells*. May 12 2021;10(5)doi:10.3390/cells10051184

15. Bhat SS, Friedmann KS, Knörck A, et al. Syntaxin 8 is required for efficient lytic granule trafficking in cytotoxic T lymphocytes. *Biochim Biophys Acta*. Jul 2016;1863(7 Pt A):1653-64. doi:10.1016/j.bbamcr.2016.04.014

16. Varrin-Doyer M, Vincent P, Cavagna S, et al. Phosphorylation of collapsin response mediator protein 2 on Tyr-479 regulates CXCL12-induced T lymphocyte migration. *J Biol Chem*. May 08 2009;284(19):13265-76. doi:10.1074/jbc.M807664200

17. Chuang HC, Wang X, Tan TH. MAP4K Family Kinases in Immunity and Inflammation. *Adv Immunol*. 2016;129:277-314. doi:10.1016/bs.ai.2015.09.006

18. Cho EC, Kuo ML, Cheng JH, et al. RRM2B-Mediated Regulation of Mitochondrial Activity and Inflammation under Oxidative Stress. *Mediators Inflamm*. 2015;2015:287345. doi:10.1155/2015/287345

19. Wang Y, Zhou Y, Graves DT. FOXO transcription factors: their clinical significance and regulation. *Biomed Res Int*. 2014;2014:925350. doi:10.1155/2014/925350

20. McDonald C, Chen FF, Ollendorff V, et al. A role for Erbin in the regulation of Nod2-dependent NF-kappaB signaling. *J Biol Chem*. Dec 02 2005;280(48):40301-9. doi:10.1074/jbc.M508538200

21. Michael M, McCormick B, Anderson KE, et al. The 5-Phosphatase SHIP2 Promotes Neutrophil Chemotaxis and Recruitment. *Front Immunol*. 2021;12:671756. doi:10.3389/fimmu.2021.671756

22. Rivers E, Thrasher AJ. Wiskott-Aldrich syndrome protein: Emerging mechanisms in immunity. *Eur J Immunol*. Nov 2017;47(11):1857-1866. doi:10.1002/eji.201646715

23. Lin J, Weiss A. The tyrosine phosphatase CD148 is excluded from the immunologic synapse and down-regulates prolonged T cell signaling. *J Cell Biol*. Aug 18 2003;162(4):673-82. doi:10.1083/jcb.200303040

24. Kretzschmar M, Doody J, Massagué J. Opposing BMP and EGF signalling pathways converge on the TGF-beta family mediator Smad1. *Nature*. Oct 09 1997;389(6651):618-22. doi:10.1038/39348

25. Parmacek MS, Solaro RJ. Biology of the troponin complex in cardiac myocytes. *Prog Cardiovasc Dis*. 2004;47(3):159-76. doi:10.1016/j.pcad.2004.07.003

26. Lehnart SE, Terrenoire C, Reiken S, et al. Stabilization of cardiac ryanodine receptor prevents intracellular calcium leak and arrhythmias. *Proc Natl Acad Sci U S A*. May 16 2006;103(20):7906-10. doi:10.1073/pnas.0602133103

27. Huang ZP, Kataoka M, Chen J, et al. Cardiomyocyte-enriched protein CIP protects against pathophysiological stresses and regulates cardiac homeostasis. *J Clin Invest*. Nov 02 2015;125(11):4122-34. doi:10.1172/JCI82423

28. Cascón A, Robledo M. MAX and MYC: a heritable breakup. *Cancer Res*. Jul 01 2012;72(13):3119-24. doi:10.1158/0008-5472.CAN-11-3891

29. Kawasaki Y, Saito T, Shirota-Someya Y, et al. Cell death-associated translocation of plasma membrane components induced by CTL. *J Immunol*. May 01 2000;164(9):4641-8. doi:10.4049/jimmunol.164.9.4641

30. Li Y, Hao H, Swerdel MR, et al. Top2b is involved in the formation of outer segment and synapse during late-stage photoreceptor differentiation by controlling key genes of photoreceptor transcriptional regulatory network. *J Neurosci Res*. Oct 2017;95(10):1951-1964. doi:10.1002/jnr.24037

31. Lin SC, Hardie DG. AMPK: Sensing Glucose as well as Cellular Energy Status. *Cell Metab*. Feb 06 2018;27(2):299-313. doi:10.1016/j.cmet.2017.10.009

32. Xu P, Ianes C, Gärtner F, et al. Structure, regulation, and (patho-)physiological functions of the stress-induced protein kinase CK1 delta (CSNK1D). *Gene*. Oct 05 2019;715:144005. doi:10.1016/j.gene.2019.144005

33. Hosono T, Tanaka T, Tanji K, Nakatani T, Kamitani T. NUB1, an interferon-inducible protein, mediates anti-proliferative actions and apoptosis in renal cell carcinoma cells through cell-cycle regulation. *Br J Cancer*. Mar 02 2010;102(5):873-82. doi:10.1038/sj.bjc.6605574

34. Kurokawa K. How is plasma calcium held constant? Milieu interieur of calcium. *Kidney Int*. Jun 1996;49(6):1760-4. doi:10.1038/ki.1996.262

35. D'Agostino S, Lanzillotta D, Varano M, et al. The receptor protein tyrosine phosphatase PTPRJ negatively modulates the CD98hc oncoprotein in lung cancer cells. *Oncotarget*. May 04 2018;9(34):23334-23348. doi:10.18632/oncotarget.25101

36. Redin E, Garrido-Martin EM, Valencia K, et al. YES1 Is a Druggable Oncogenic Target in SCLC. *J Thorac Oncol*. Dec 2022;17(12):1387-1403. doi:10.1016/j.jtho.2022.08.002
